# What’s in a face: Automatic facial coding of untrained study participants compared to standardized inventories

**DOI:** 10.1371/journal.pone.0263863

**Published:** 2022-03-03

**Authors:** T. Tim A. Höfling, Georg W. Alpers, Björn Büdenbender, Ulrich Föhl, Antje B. M. Gerdes

**Affiliations:** 1 Department of Psychology, School of Social Sciences, University of Mannheim, Mannheim, Germany; 2 Business School, Pforzheim University of Applied Sciences, Pforzheim, Germany; Aristotle University of Thessaloniki, GREECE

## Abstract

Automatic facial coding (AFC) is a novel research tool to automatically analyze emotional facial expressions. AFC can classify emotional expressions with high accuracy in standardized picture inventories of intensively posed and prototypical expressions. However, classification of facial expressions of untrained study participants is more error prone. This discrepancy requires a direct comparison between these two sources of facial expressions. To this end, 70 untrained participants were asked to express joy, anger, surprise, sadness, disgust, and fear in a typical laboratory setting. Recorded videos were scored with a well-established AFC software (FaceReader, Noldus Information Technology). These were compared with AFC measures of standardized pictures from 70 trained actors (i.e., standardized inventories). We report the probability estimates of specific emotion categories and, in addition, Action Unit (AU) profiles for each emotion. Based on this, we used a novel machine learning approach to determine the relevant AUs for each emotion, separately for both datasets. First, misclassification was more frequent for some emotions of untrained participants. Second, AU intensities were generally lower in pictures of untrained participants compared to standardized pictures for all emotions. Third, although profiles of relevant AU overlapped substantially across the two data sets, there were also substantial differences in their AU profiles. This research provides evidence that the application of AFC is not limited to standardized facial expression inventories but can also be used to code facial expressions of untrained participants in a typical laboratory setting.

## Introduction

Emotional experiences encompass a multitude of bodily changes and most salient among them are emotional facial expressions [1, 2]. The study of emotional facial expressions has received wide attention [3], because they are linked to internal states of a person [4]. Researchers typically use observational techniques to classify specific emotional facial expressions [5, 6]; the most prominent method is the Facial Action Coding System (FACS; [7]). It defines relevant facial movements as Action Units (AU), which are indicative of specific emotional facial expressions. Although FACS has proven to be a very useful, valid, and reliable system, its application to quantify a large array of facial configurations is rather laborious.

Recent advances in computer vision technology enable researchers to automatically measure facial activity in dynamic videos or static photos [8, 9]. In comparison to human FACS coding, automatic facial coding (AFC) offers several advantages: it is dramatically more time efficient because it can analyze a large number of facial expressions without human effort [10]. Moreover, AFC is less intrusive and less susceptible to motion artifacts [11], but also less sensitive to more subtle facial responses compared to psycho-physiological measures like electromyography [12, 13].

AFC extracts movement from transient facial features (i.e., AU activity), its scores correspond well with those from trained human FACS coders [14–16]. In addition to the measurement of AU activities, AFC software operates with machine learning procedures that are trained to classify different emotion categories. Therefore, AFC integrates AU profiles into a probability estimate of specific emotional facial expressions. The categories for such supervised machine learning typically include six basic emotions which are prominent in psychological research, i.e., joy, surprise, anger, sadness, disgust and fear [17].

In order to test AFC validity, previous research has typically used highly standardized static [18–21] and dynamic facial expressions [22–24]. These studies show that AFC has good to excellent sensitivity and specificity for the intended emotion categories. Importantly, actors in such standardized inventories are trained to display prototypical facial expressions. Only few studies have also tested the validity of AFC in more naturalistic facial expressions of untrained participants who posed facial expressions. Two studies documented that AFC is sensitive for posed joy and anger, but with larger sensitivity for joyful compared to angry faces [13, 18]. Two other studies, in which participants posed all six emotions, reported substantial differences in sensitivity for specific emotion categories [21, 25]. One study also showed a substantial drop in accuracy for sad facial expressions and almost no specific detection of fearful faces [21]. The other demonstrated misclassifications of angry and disgusted faces [25]. Taken together, the sparse number of studies on the validity of AFC in posed basic emotional facial expressions of untrained participants, show that joy faces can be classified more accurately than other categories and that there is substantial variation in particular, between unpleasant emotion categories.

Hence, AFC classifies emotional facial expressions from standardized pictures very well. However, performance is much more variable in facial expressions of untrained participants. This discrepancy between results from standardized or non-standardized expressions is problematic if AFC is to be used to quantify emotional facial expressions in real life or in a typical laboratory setting. Machine learning procedures underlying the AFC software were trained with emotional facial expressions from standardized inventories, they may therefore be best suited to classify prototypical facial expressions. When untrained study participants do not display prototypical facial expressions or display them with less intensity [26, 27] these algorithms may not be as successful.

In order to evaluate the generalizability of AFC to measure emotional facial expressions, the present study directly compares the sensitivity of AFC for posed facial expressions in data from untrained participants and in standardized picture inventories. First, we compare these two sources of emotional facial expressions based on emotion scores (i.e., probability estimates of specific emotion categories). Second, we identified relevant subsets of AU with a new machine-learning approach. We developed a machine-learning classifier that distinguishes between neutral and emotional faces separately for emotion categories and datasets. In order to identify relevant AU subsets for a specific emotion category we calculated variable importance information for all AUs. Third, we compare the AU profiles in order to estimate intensity as well as profile differences between datasets. This will provide important information about the validity of AFC as a research tool.

## Method

The University Mannheim Research Ethics Committee approved the experiment (EK Mannheim09-3/2018). We obtained written consent from our participants.

### Facial expressions of trained actors

We analyzed pictures of 70 female actors selected from three well-known picture inventories: The Karolinska Directed Emotional Faces [28], the Warsaw Set of Emotional Facial Expression pictures [29] and the Radboud Faces Database [30]. All actors display six basic emotions (joy, anger, surprise, sadness, disgust, and fear) as well as a neutral facial expression. The software was not able to detect the face in the pictures of one actor as well as in pictures of joy and neutral of two other actors, and consequentially, no data are available for these facial expressions (dropout = 2.2%).

### Facial expressions of untrained participants

#### Participants

We recorded videos of 70 undergraduate female students who participated in the experiment and actively expressed basic emotional facial expressions cued with presented pictures of emotional facial expressions. General exclusion criteria were age under 18, use of acute psychoactive medication, acute episode of a mental disorder, or severe somatic disease, as well as wearing glasses. Participants with corrected-to-normal vision were asked to wear contact lenses during the experiment. One participant had to be excluded due to technical failure. All participants received a compensation of either 8€ or student course credit and they signed informed consent before the data collection.

#### Stimulus material, apparatus and procedure

High-precision software (Presentation Tool; Version 3; Noldus Information Technology) was used for presentation of the pictures (i.e., cues). Pictures were shown centrally on a 21-inch monitor with a resolution of 1024x768. Videos of participants’ faces were recorded with a Logitec HDC 615 video camera, which was placed above the computer screen (15fps, 1920x1080) at approximately a distance of 70 cm. Picture cues with emotional facial expressions were presented to the participants for 5 s in randomized order with a visual angle of 17.5° x 26.1°. Participants were instructed to actively express the presented emotional facial expressions as soon and as long as the pictures were presented. There was an inter-trial-interval with randomized durations (*M* ~ 3344 ms, *SD* ~ 18 ms).

We selected 70 photographs from the Radboud Faces Database as stimuli for the participants ([30]; model numbers: 01, 02, 04, 08, 12, 14, 19, 31, 32, 56). Each of the ten actors presented neutral, joyful, angry, sad, disgusted, scared and surprised facial expression with frontal face and directed gaze. We exclusively selected pictures from females because previous research indicated that they elicit stronger emotional reactions [31]. In order to avoid confusions due to recognition errors, we labeled each picture with the intended emotion word. Participants were familiarized with the task through practice trials which preceded the main experimental block. Pictures of two models served as practice trials and all other 56 pictures served as experimental trials.

### Measurement preprocessing

Picture frames of both datasets were processed with FaceReader software (FR; Version 7.1, Noldus Information Technology) and aggregated with Observer XT offline (Version 12.5, Noldus Information Technology). FR analyzes facial configurations in two subsequent steps to estimate AU activity and emotion scores [32]. Exported FR parameters included 20 AU (AU01 Inner Brow Raiser, AU02 Outer Brow Raiser, AU04 Brow Lowerer, AU05 Upper Lid Raiser, AU06 Cheek Raiser, AU07 Lid Tightener, AU09 Nose Wrinkler, AU10 Upper Lid Raiser, AU12 Lip Corner Pull, AU14 Dimpler, AU15 Lip Corner Depressor, AU17 Chin Raiser, AU18 Lip Puckerer, AU20 Lip Stretcher, AU23 Lip Tightener, AU24 Lip Pressor, AU25 Lips Part, AU26 Jaw Drop, AU27 Mouth Stretch and AU43 Eyes Closed) as well as the above mentioned FR emotion scores (FR Joy, FR Anger, FR Sadness, FR Disgust, FR Fear and FR Surprise). All FR parameters were multiplied by 100 to improve readability of results.

In order to improve comparability between photos of trained actors and videos of untrained participants, we took the following measures: First, in contrast to the pictures of trained actors, AFC parameters of the untrained participants were baseline-corrected for each trial–i.e., mean activations of the second before stimulus onset (baseline) were subtracted from the following activity–to account for artefacts caused by a different video angle. Second, AFC parameters of untrained participants were averaged for the most active time interval per trial (second 3 to 5 after stimulus onset). S1 Appendix in S1 File shows averaged and uncorrected FR Scores time courses of exemplary trials for each emotion category. These data demonstrate that untrained participants display facial expressions with constant intensities for this time interval. In addition, a trained FACS coder inspected randomly selected 3 trials from each participant and verified that they constantly held the expression until the end of the trial as instructed. Importantly, the software processes single frames (photos) or multiple frames (videos) in the same technical way (see paragraph above).

### Selection of action units

In order to identify relevant AUs involved in the expression of a certain emotion category, we implemented a machine learning procedure [33]. We trained twelve (six emotion categories x two datasets) independent multi-layer perceptrons, a basic form of artificial neural network, to distinguish the intended emotion from neutral facial expressions. Our machine learning procedure involved the following sequential steps: preprocessing, hyperparameter tuning and evaluation of the model performance in a grouped 5-fold cross-validation. All analyses were conducted with the caret R-package [34], which utilizes the multi-layer perceptron algorithm from the RSNNS R-package [35]. In the preprocessing step, we removed all near zero-variance features and applied min-max normalization to the remaining predictors.

We tuned the hyperparameter number of nodes (i.e., neurons) in the single hidden-layer with an extensive search (range [1:*n*]), where *n* is the number of AU without near zero variance. This procedure was applied to maximize the average accuracy and minimize the number of neurons necessary. This means for each target emotion *n* models are trained with one to *n* neurons in the hidden-layer. From the resulting array of models, the one with the highest average accuracy in the cross-validation was chosen. If multiple models achieved identical average accuracies, the one with the lowest number of neurons necessary was chosen. Higher number of neurons in the hidden-layer typically indicates higher complexity of the model. However, we did not observe a large drop in accuracy by changing the optimal number of neurons and, hence, advise a careful interpretation of the number of neurons in terms of model complexity. More information on the machine learning procedure can be obtained from S2 Appendix in S1 File.

All models reached very good to excellent average accuracies (> 90%) and Cohen’s *κ* scores (> .80; [36]) in the 5-fold cross-validation. Afterwards we determined the relative variable importance of an AU for the binary classification of a target emotion in each of the twelve models with the IML R-package [37] in order to identify relevant AU for a specific emotion category. AU importance was quantified with the model-agnostic permutation feature importance [38]. We included an AU in the further analysis if it was important in at least one of the two datasets and reached a permutation importance value over 0.025.

### Data reduction and analysis

The averages of FR measures were calculated separately for the trained actors and untrained participants (*Dataset*) of each *Emotion* (neutral, joyful, angry, sad, disgusted, scared, and surprised). In order to compare intensity levels of the FR emotion scores between both datasets and all emotion categories, analyses of the FR emotion scores were conducted for each *Emotion* separately. We calculated ANOVAs for the corresponding FR emotion scores separately for each emotion category (i.e., FR Joy for intended joy facial expressions, FR Anger for intended anger facial expressions etc.) resulting in a 2 (*Dataset*) x 6 (*Emotion*) design. Furthermore, we calculated independent post-hoc *t*-tests between both datasets separately for each FR score. Afterwards we analyzed differences of the AU profiles between the datasets with parallelism tests of the profile analyses (i.e., the variant of MANOVA using Hotelling’s *T*^2^; [39]) regarding the factors *Dataset* and *AU*. In order to avoid biased effect sizes, we only included relevant AU for specific emotion categories obtained from the machine learning based variable selection procedure. When differences in the profiles between datasets were significant (interaction effect between *Dataset* and *AU)*, we calculated independent post-hoc *t*-tests between both datasets separately for each AU. Eta-squared (*η*^*2*^) was reported as effect size for *F*-tests [40] (*η*_*p*_^2^ ≥ .01 small; *η*_*p*_^2^ ≥ .06 medium; *η*_*p*_^2^ ≥ .14 large; [41]). Cohen’s d was reported for *t*-tests and interpreted according to Cohen [42] and Sawilowsky [43] (*d* ≥ 0.2 small; *d* ≥ 0.5 medium; *d* ≥ 0.8 large; *d* ≥ 1.2 very large; *d* ≥ 2.0 huge). Bonferroni-Correction for multiple post-hoc *t*-tests was applied.

## Results

### AFC emotion scores

**Fig 1:**
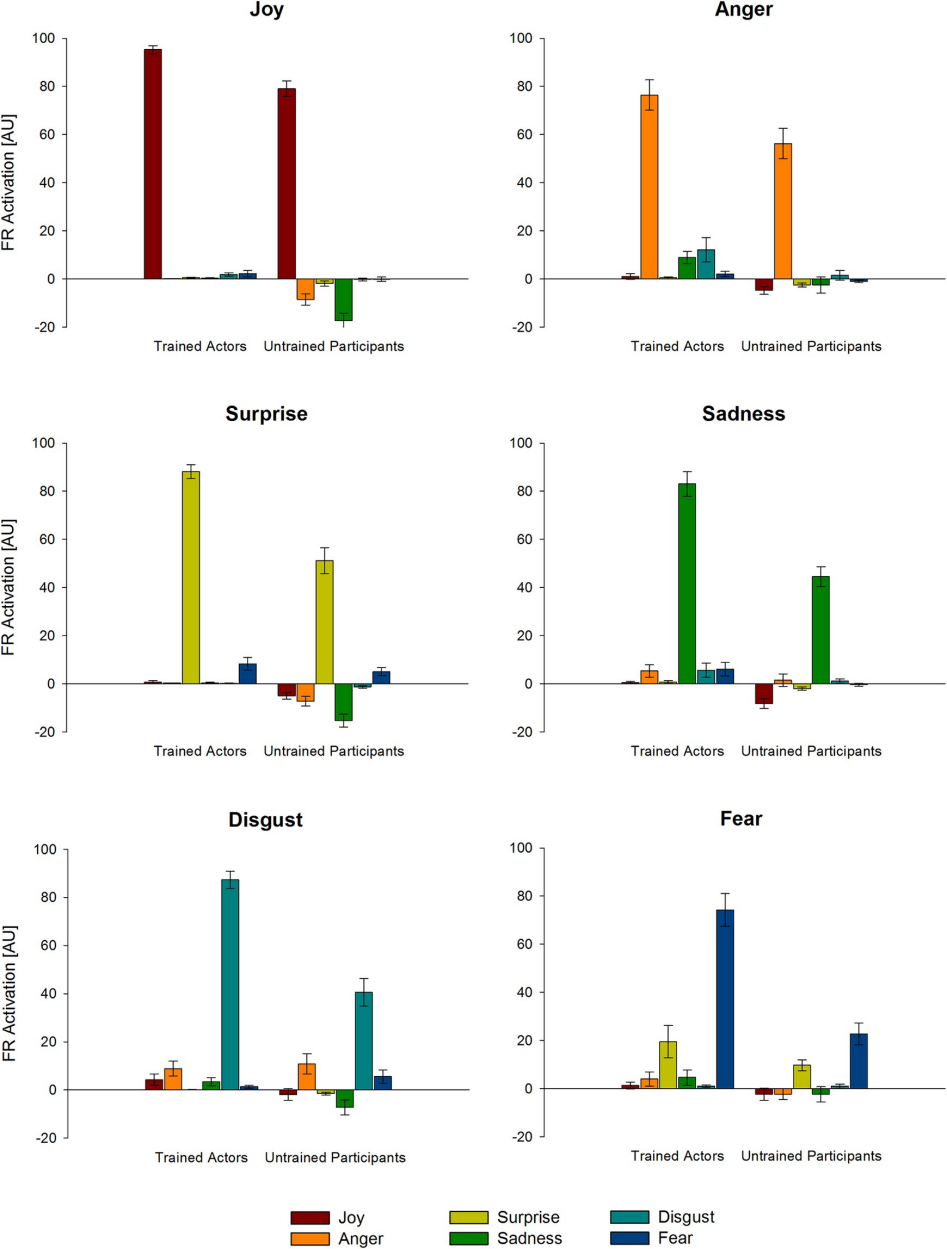
FaceReader (FR) emotion scores. Mean FR emotion scores separately for trained actors and untrained participants in arbitrary units [AU]. Note. Panel titles refer to the intended emotional facial expressions. The colored bars indicate the different emotion scores measured by the software. Error bars indicate 95% confidence intervals.

We analyzed FR emotion scores for all six emotion categories and the interaction between trained actors and untrained participants. We found a significant interaction effect between *Emotion* and *Dataset*, *F*(5, 670) = 20.78, *p* < .001, *η*_*p*_^2^ = .13, as well as a significant main effect for *Emotion*, *F*(5, 670) = 64.71, *p* < .001, *η*_*p*_^2^ = .33, and a significant main effect for *Dataset*, *F*(1, 134) = 332.54, *p* < .001, *η*_*p*_^2^ = .71 (see Fig 1). Intensities of the FR emotion scores for the displayed emotional facial expressions showed stronger differences between emotion categories for the untrained participants *F*(5, 340) = 71.90, *p* < .001, *η*_*p*_^2^ = .51, compared to the trained actors *F*(5, 330) = 11.64, p < .001, *η*_*p*_^2^ = .15. This interaction was followed up by comparisons of both datasets for each emotion category.

Trained actors always expressed emotions with significantly higher intensities compared to untrained participants (see Table 1): The difference between datasets was moderate for the expression of anger, very large for joy and sadness and huge for surprise, fear and disgust. Effect sizes for differences to zero for all FR emotion scores and emotion categories can be obtained in S3 Appendix in S1 File and FR Valence measures can be obtained in S4 Appendix in S1 File.

**Table 1:** Mean differences of corresponding FaceReader emotion scores (FR) between data from untrained participants’ and trained actors’ emotional facial expressions in arbitrary units.

Emotion Category	Untrained Participants *M*, (*SD*)	Trained Actors *M*, (*SD*)	*t*	*df*	*p*	*d*	Effect Interpretation
Joy	79.02 (13.46)	95.27 (6.91)	8.90	102.18	< .001	1.52	Very Large
Anger	56.22 (25.95)	76.36 (26.31)	4.53	136	< .001	0.77	Moderate
Surprise	51.15 (22.17)	88.19 (11.96)	12.21	104.50	< .001	2.08	Huge
Sadness	44.50 (17.38)	83.01 (21.16)	11.68	136	< .001	1.99	Very Large
Disgust	40.55 (23.94)	87.32 (15.09)	13.73	114.69	< .001	2.34	Huge
Fear	22.71 (19.20)	74.25 (28.60)	12.43	118.95	< .001	2.12	Huge

*Note*. t = t-values, df = corrected degrees of freedom, p = p-values, d = Cohen’s d. M and SD represent mean and standard deviation. *d* ≥ 0.2 small; *d* ≥ 0.5 medium; *d* ≥ 0.8 large; *d* ≥ 1.2 very large; *d* ≥ 2.0 huge.

### Relevance of specific action units

In order to determine significant variation in the AU profiles between untrained participants and trained actors, we identified relevant active AU subsets for each emotion category. We first trained artificial neural networks (multi-layer perceptrons; [35]) in both datasets to binary classify between a target emotion category and neutral expression based on the AU activation. All emotion categories were classified with high accuracy for untrained participants and trained actors (> 90%; see Table 2 for more details).

**Table 2:** Performance metrics for the twelve multi-layer perceptrons to classify between neutral and emotional facial expressions separately for untrained participants and trained actors.

	Untrained Participants				Trained Actors			
Emotion Category	Neurons	Accuracy	Kappa	*F*	Neurons	Accuracy	Kappa	*F*
Joy	1	1.00	1.00	1.00	1	.993	.985	.993
Anger	2	.956	.912	.954	2	.993	.986	.993
Surprise	1	.972	.944	.970	1	.971	.825	.972
Sadness	14	.918	.836	.910	1	.913	.943	.909
Disgust	18	.978	.956	.976	1	.993	.986	.994
Fear	2	.970	.940	.968	2	.972	.944	.974

*Note*. Performance of twelve multi-layer perceptrons (MLP) in the contrasted datasets (only trials of one target emotion and neutral trials). Neurons refer to the number of nodes in the single hidden-layer of the MLP and represents a hyperparameter of the model. Performance metrics (accuracy, kappa scores, F_1_) are averaged over all five folds.

**Fig 2:**
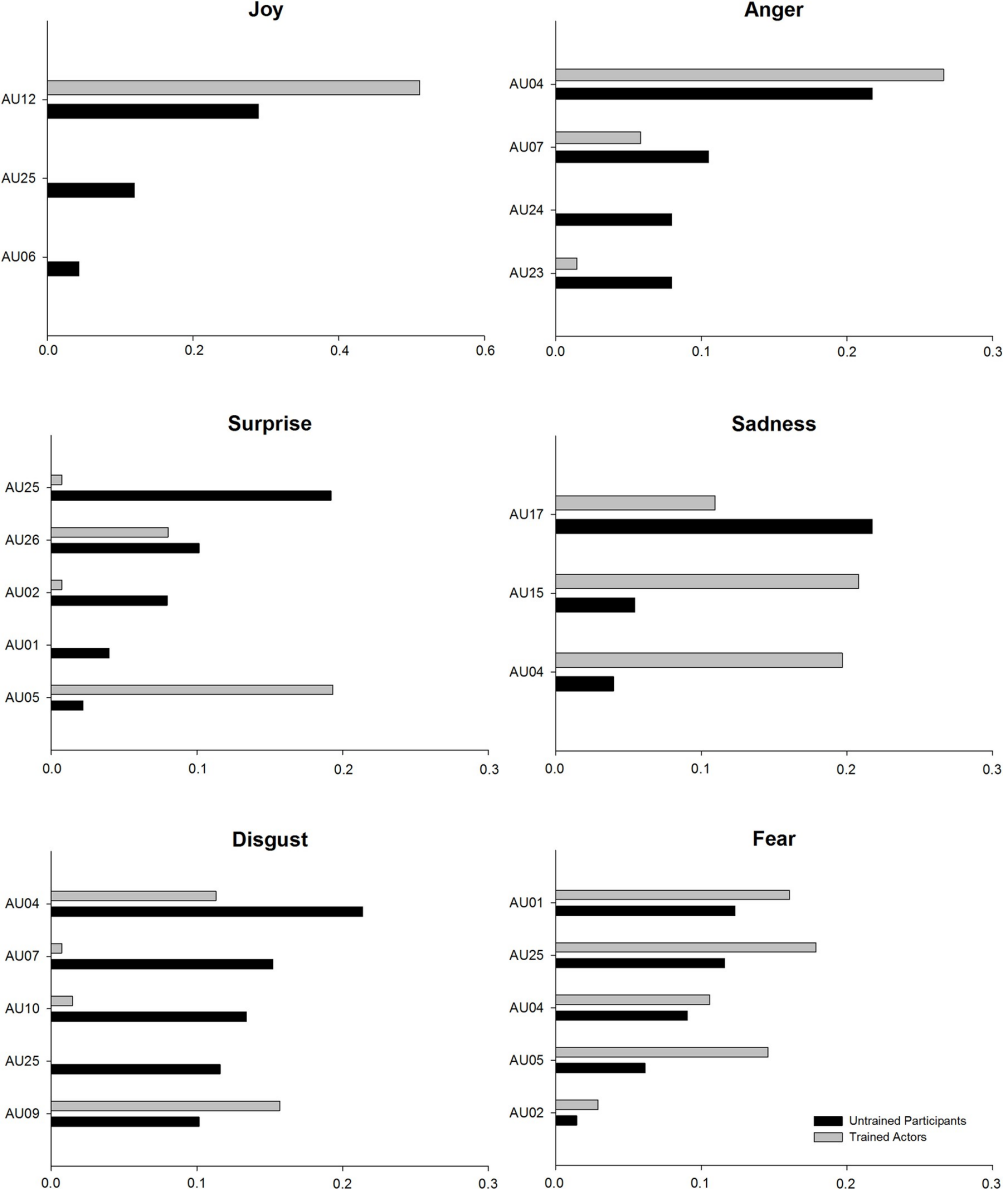
Variable importance of action units. Note. Bars indicate Variable Importance (VI) Score of an Action Unit (AU) for the binary classification of an intended emotion against neutral facial expression separately for trained actors’ and untrained participants’ datasets. AU with VI score below 0.025 in both datasets are considered irrelevant for classification. Panels titles refer to the intended emotional facial expressions.

**Fig 3:**
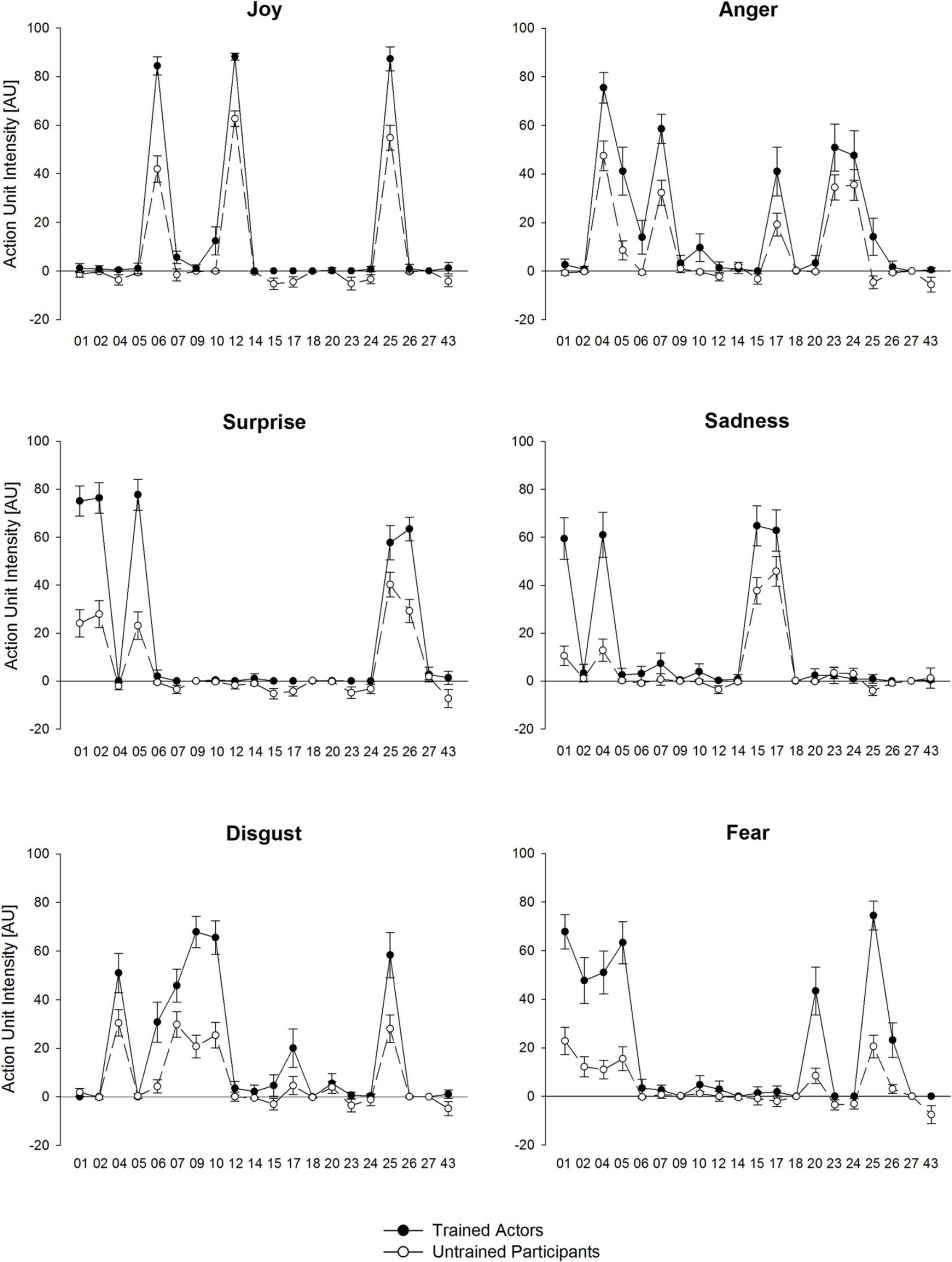
Action unit profiles. Mean action unit (AU) intensity trained actors and untrained participants measured by FaceReader in arbitrary units [AU]. Note. Panels titles refer to the intended emotional facial expressions. Error bars indicate 95% confidence intervals.

Next, we used permutation variable importance [38], a model-agnostic approach, to rank the importance of the predictors (i.e., AUs) for each artificial neural network to identify relevant AU subsets separately for each emotion category (see also Fig 2). This variable selection procedure corresponds well with descriptive AU profiles for facial expressions of untrained participants (see Fig 3 and S5 Appendix in S1 File). However, variable importance for facial expressions of trained actors indicates a substantially reduced set of necessary AUs for classification of most emotion categories. This is probably because of a lower variance in the AU activity, as can be seen in Table B1 (S2 Appendix in S1 File). For example, the artificial neural network the activity of AU12 (lip corner pull) is sufficient to distinguish between neutral and joyful facial expressions of trained actors, whereas the AU profiles for trained actors (Fig 3) clearly shows a coactivation of AU06 and AU12. In addition, we included AU01 to the relevant AU subset in sad faces for the following profile analysis, because trained actors strongly activated this AU in this specific emotion category. Hence, the following sets of relevant AUs were included in subsequent analyses: joy (AU06, 12, 25), anger (AU04, 07, 23, 24), surprise (AU01, 02, 05, 25, 26), sadness (AU01, 04, 15, 17), disgust (AU04, 07, 09, 10, 25) and fear (AU01, 02, 04, 05, 25).

### Action unit profiles

Analysis of the AU subsets revealed strong multivariate interactions between *Dataset* and *AU* for most of the emotion categories which indicates differences in the AU profiles between untrained participants and trained actors (see Fig 3).

As reported in Table 3, interaction effects were large for joy, surprise, sadness, and disgust, and moderate for fear and anger. Beside significant interactions, all emotion categories show a large main effect with overall lower AU intensities for untrained participants compared with trained actors, correspondingly to the previously reported effects for FR Scores. For instance, compared to joy, surprise, disgust and fear (*η*_*p*_^2^ ≥ .53), sadness (*η*_*p*_^2^ = .36) and in particular anger (*η*_*p*_^2^ = .17) showed reduced main effects of the overall AU activity between untrained participants and trained actors.

**Table 3:** MANOVA for specific Action Unit (AU) activity and datasets (untrained participants’ and trained actors’ emotional facial expressions).

	*Dataset x AU*				*Dataset*				*AU*			
Emotion Category	*dfs*	*F*	*p*	*η*_*p*_^2^	*dfs*	*F*	*p*	*η*_*p*_^2^	*dfs*	*F*	*p*	*η*_*p*_^2^
Joy	2,133	15.52	< .001	.19	1,134	228.76	< .001	.63	2,133	31.93	< .001	.32
Anger	3,134	2.95	.035	.06	1,136	27.40	< .001	.17	3,134	20.86	< .001	.32
Surprise	4,133	9.58	< .001	.22	1,136	240.22	< .001	.64	4,133	1.91	.112	.05
Sadness	3,134	10.22	< .001	.19	1,136	77.85	< .001	.36	3,134	15.41	< .001	.26
Disgust	4,133	10.80	< .001	.25	1,136	154.61	< .001	.53	4,133	2.85	.027	.08
Fear	4,133	4.68	< .001	.12	1,136	199.38	< .001	.59	4,133	29.59	< .001	.47

*Note*. dfs = degrees of freedom, F = F-Values, p = p-values, η_p_^2^ = partial eta squared. AU subsets: Joy (AU6, AU12, AU25), Anger (AU4, AU7, AU23, AU24), Surprise (AU1, AU2, AU5, AU25, AU26), Sadness (AU1, AU4, AU15, AU17), Disgust (AU4, AU7, AU9, AU10, AU25), Fear (AU1, AU2, AU4, AU5, AU25).

In order to resolve the interaction patterns, we calculated post hoc comparisons between both datasets for specific AU (see Table 4). For joy, differences between untrained participants and trained actors are stronger pronounced for AU06 and AU12 compared to AU25. For anger, differences between untrained participants and trained actors are stronger pronounced in the eye region (AU04 and AU07) compared to the mouth region (AU23 and AU24). For surprise, untrained participants and trained actors showed very large to huge differences for AUs from the upper and lower face (AU01, AU02, AU05, and AU26). For sadness, untrained participants expressed sadness mainly with their mouth region (AU15 and AU17) and less with the eye region (AU01 and AU04) in comparison to trained actors. It is notably that untrained participants expressed sadness mainly with mouth region (AU15 and AU17), whereas trained actors also moved their eyebrows (AU01 and AU04; see also S5 Appendix in S1 File). For disgust we observed stronger differences in the mouth region (AU10 and AU25) and in particular for the nose region (AU09) between both datasets. For fear, untrained participants and trained actors differed more in the mouth region (AU25) compared to the eye region (AU01, AU02, AU04, and AU05).

**Table 4:** Mean differences of Action Unit (AU) activity between data from untrained participants’ and trained actors’ emotional facial expressions in arbitrary units.

Emotion Category	AU	Untrained Participants (*M*, *SD*)	Trained Actors (*M*, *SD*)	*t*	*df*	*p*	*d*	Effect Inter-pretation
Joy	06	41.90 (22.91)	84.39 (15.49)	12.70	119.77	< .001	2.17	Huge
	12	62.63 (13.36)	88.15 (6.00)	14.43	94.97	< .001	2.46	Huge
	25	54.79 (21.05)	87.31 (22.21)	9.19	133.98	< .001	1.50	Very Large
Anger	04	47.46 (25.64)	75.49 (25.90)	6.39	135.99	< .001	1.09	Large
	07	32.26 (21.39)	58.53 (25.10)	6.62	132.67	< .001	1.13	Large
	23	34.43 (21.47)	50.85 (40.35)	2.99	103.65	< .001	0.51	Moderate
	24	35.47 (26.45)	47.57 (42.51)	2.01	113.79	< .001	0.34	Small
Surprise	01	24.10 (23.62)	75.05 (26.48)	11.93	136	< .001	2.03	Huge
	02	27.85 (23.42)	76.31 (26.57)	11.36	136	< .001	1.93	Very Large
	05	23.08 (23.77)	77.66 (26.83)	12.65	136	< .001	2.15	Huge
	25	40.18 (21.41)	57.68 (29.93)	3.95	123.15	< .001	0.67	Moderate
	26	29.19 (19.91)	63.37 (20.51)	9.93	135.88	< .001	1.69	Very Large
Sadness	01	10.52 (16.64)	59.40 (36.38)	10.15	95.25	< .001	1.73	Very Large
	04	12.82 (19.29)	60.92 (38.91)	9.20	99.51	< .001	1.57	Very Large
	15	37.68 (22.91)	64.74 (34.66)	5.41	117.88	< .001	0.92	Large
	17	45.74 (25.65)	62.80 (35.94)	3.21	123.02	.002	0.55	Moderate
Disgust	04	30.38 (22.59)	52.30 (33.90)	4.47	118.44	< .001	0.76	Moderate
	07	29.73 (21.91)	46.86 (27.75)	4.02	136	< .001	0.69	Moderate
	09	20.72 (19.18)	70.41 (25.85)	12.82	136	< .001	2.18	Huge
	10	25.32 (22.04)	68.48 (27.49)	10.17	136	< .001	1.73	Very Large
	25	28.04 (23.68)	61.09 (38.92)	6.03	112.28	< .001	1.03	Large
Fear	01	22.75 (23.26)	67.77 (29.34)	9.99	136	< .001	1.70	Very Large
	02	12.11 (17.42)	47.65 (39.36)	6.86	93.66	< .001	1.17	Large
	04	10.98 (16.18)	50.94 (36.71)	8.28	93.46	< .001	1.41	Very Large
	05	15.46 (20.37)	63.25 (35.89)	9.62	107.70	< .001	1.64	Very Large
	25	20.51 (19.24)	74.37 (24.60)	14.33	128.52	< .001	2.44	Huge

*Note*. t = t-values, df = corrected degrees of freedom, p = p-values, d = Cohen’s d. M and SD represent mean and standard deviation. *d* ≥ 0.2 small; *d* ≥ 0.5 medium; *d* ≥ 0.8 large; *d* ≥ 1.2 very large; *d* ≥ 2.0 huge.

## Discussion

Technological advances have only recently enabled machines to read facial expressions. In this study we directly compared state-of-the-art Automatic Facial Coding (AFC) measures of emotional facial expressions generated by untrained participants in a typical laboratory setting and prototypical facial expressions from standardized inventories (i.e., trained actors). Untrained participants compared to trained actors showed substantially less intense facial expressions which is in line with previous research [26, 27]. Our present study indicates that most emotion categories, in particular joyful faces, can be detected with both high sensitivity and specificity. One exception is the detection of fearful faces of untrained participants which are detected with much lower sensitivity and specificity compared to those of trained actors. Although profiles of relevant AU overlapped substantially across the two data sets, we also observed several differences in the relative intensity and shaping of the AU profiles depending on the specific emotion expressed which replicates and also extends several prior findings of the current AFC literature. Importantly, the present study supports AFC as a valuable research tool to detect intense emotional facial expressions of untrained study samples.

AFC is more sensitive to detect joyful facial expressions compared to unpleasant facial expressions for standardized picture inventories [20–22, 44] as well as for untrained participants who are prompted to mimic such facial expressions [18, 21, 25]. Emotion scores of unpleasant emotions retrieved from the untrained sample in our study are very similar to scores reported by Stöckli and colleagues [21], but in contrast, we found sadness scores in sad faces were more pronounced than disgust scores in disgusted faces. In comparison to the mimicking condition of Sato and colleagues [25] we found more variation in the sensitivity to different unpleasant facial expressions but also more specificity regarding other emotion scores for most of the emotion categories as this study reported stronger misclassifications.

We also observed some noteworthy differences between AU profiles of expressions posed by trained actors’ and untrained participants. Although underlying artificial networks were accurate in classification of emotion categories for both samples, fewer AUs were important for facial expressions of the trained actors compared to the untrained participants. Specifically, for the untrained participants, this method corresponds with patterns of the AU profiles and therefore appears to be a promising new method to determine relevant AU in facial expression research. Regarding the AU subsets for specific emotion categories, trained actors also used the cheek raiser (AU6) to express disgust and the upper lid raiser (AU5) to express anger which was not observed in the sample of untrained participants. Furthermore, while sadness was expressed with AU activity around the eyes and lips in trained actors, our untrained participants mainly expressed sadness with the lip corner depressor (AU15) and chin raiser (AU17) and only to a moderate extend with movements of the upper face (AU1 and AU4). These findings clearly demonstrate that trained actors and untrained participants express the same emotions differently.

### Limitations and outlook

In fact, prototypical facial expressions (i.e., expressions of trained actors in the present study) are recognized by AFC much more clearly than more naturalistic emotional facial expressions [27]. However, AFC accuracy of such prototypical facial expressions does not directly correspond with accuracy of analyses in naturally occurring emotional facial reactions. The present findings demonstrate how trained and untrained emotional expression differ in intensity as well as the profile of AUs. Thus, clearly limits ecological validity of previously reported accuracies of AFC. Nevertheless, trained actors who display intense prototypical facial expressions according to FACS instructions are generally been used to validate AFC systems; limiting ecological validity of previously reported accuracies. Instead, untrained emotional facial expressions are a better benchmark for ecological validity of AFC in emotion research. More data like ours is necessary to establish the application of AFC in emotion research.

Our design may still overestimate AFC performance compared to real-life situations because we instructed our participants to pose facial expressions and presented them picture cues for the emotional expressions that they mimicked [45, 46]. Although this is an established experimental paradigm in facial expression research [18, 21], it clearly intensified facial expressions [25]. Visually presented pictures of emotional faces elicit emotional reactions [4, 47], but they do not elicit high levels of arousal [48] and the facial response can vary depending on the different picture inventories [49]. In order to account for such biases, future studies should consider analysis of entirely spontaneous emotional facial expressions, as they can be observed when people imagine emotional situations or when they perceive emotional stimuli such as sounds or visual scenes [12]. Accordingly, more naturalistic research settings have to be approached in future studies [50]. Until further technological progress is made, AFC may not yet be capable of detecting very subtle emotional facial expressions in contrast to other research methods like EMG [13].

Generalizability of machine learning procedures could be improved if algorithms were not exclusively trained on prototypical facial expressions (i.e., from standardized material), but also incorporated more naturalistic facial expressions. In particular, if an AFC procedure is trained with highly standardized material, accuracy rates could be inflated and might not generalize well to the response of a typical study participant. Hence, studies that use standardized material in order to validate a specific AFC procedure instead of more naturalistic sources should be interpreted with caution. However, with regards to the FaceReader software, which was used in the present study, emotion scores corresponded well with intensities of relevant AUs and hence, robustly reflected intensities of the software-generated emotion scores also in untrained participants.

While AFC is thought to generate reproducible results for similar faces, human face perception is highly specialized [51] and can be substantially influenced by goals [52], internal states [53], or perceived relevance to the observer [54]. AFC is context independent which is a great advantage in terms of an objective research tool on the one hand. On the other hand, interpreting the meaning of facial expressions often depends on the specific context and situation of a person which needs to be taken into account in future developments in this research area and hence, should be combined with other meaningful non-verbal expression channels like voice or gesture recognition tools.

## Conclusion

The present study clearly demonstrates that AFC can be used as a research tool to detect intense emotional facial expressions. At its current state, it accurately extracts information from facial expressions of basic emotions in standardized material (i.e., established picture inventories). Also, generalizability of AFC’s validity to detect emotional facial expressions of untrained participants in a typical laboratory setting is high for most emotion categories. However, we reported specific differences in AU profiles between expressions of trained actors and untrained participants. This has important implications for the development of future AFC systems. They clearly need to be fine-tuned to detect more naturalistic facial expressions and more research is needed on the validity of AFC for spontaneous emotional facial expressions. Nevertheless, we expect that this novel research method will be very useful for a realm of applications and theoretical perspectives.

## Supporting information

S1 File (PDF)
