# Supplementary material for: What’s in a face: Automatic facial coding of untrained study participants compared to standardized inventories
Source: PLoS One. 2022 Mar 3;17(3):e0263863. doi: 10.1371/journal.pone.0263863 (PMC8893617; doi:10.1371/journal.pone.0263863)
Supplement: S1 File — (PDF) [file pone.0263863.s001.pdf]

S1 Appendix

Baseline-Uncorrected Mean Activation Courses of FaceReader Emotion Scores

Figure A1. Intended Joy Facial Expressions

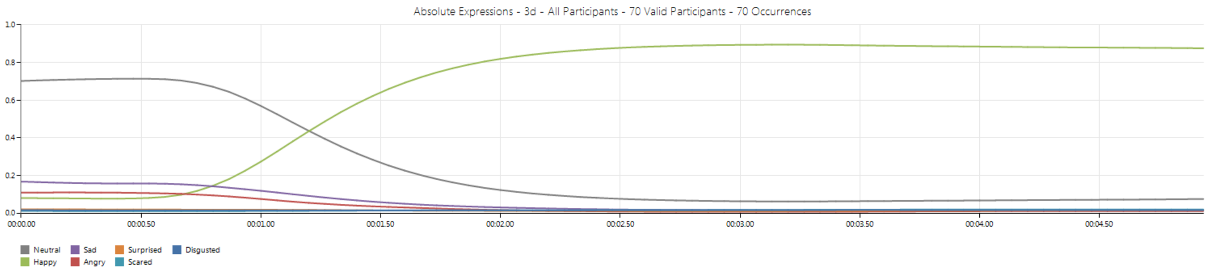

Figure A2. Intended Anger Facial Expressions

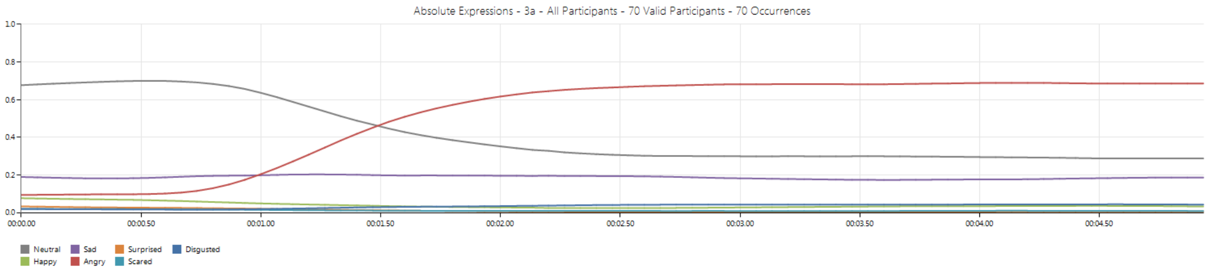

Figure A3. Intended Surprise Facial Expressions

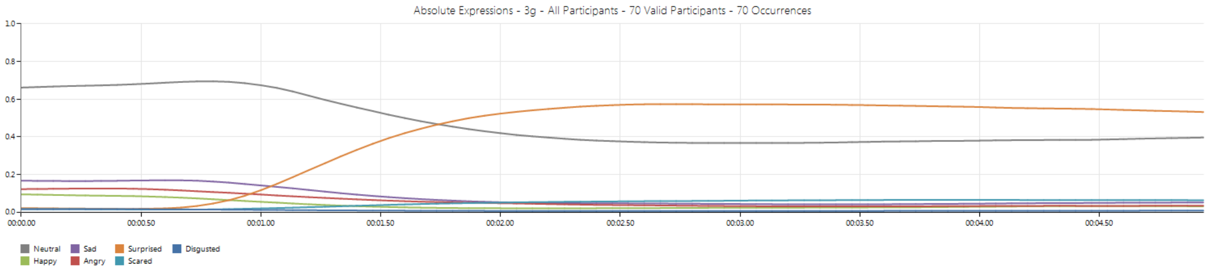

Figure A4. Intended Sadness Facial Expressions

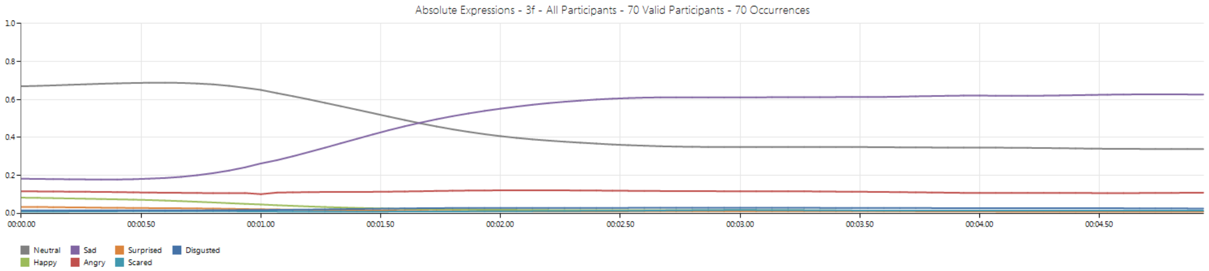

Figure A5. Intended Disgust Facial Expressions

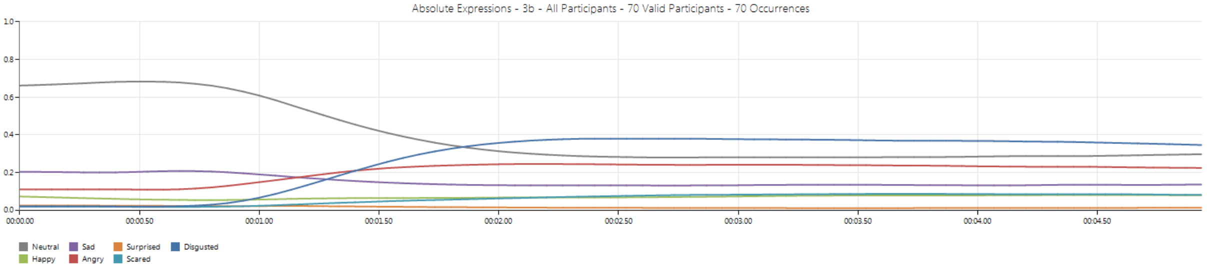

Figure A6. Intended Fear Facial Expressions

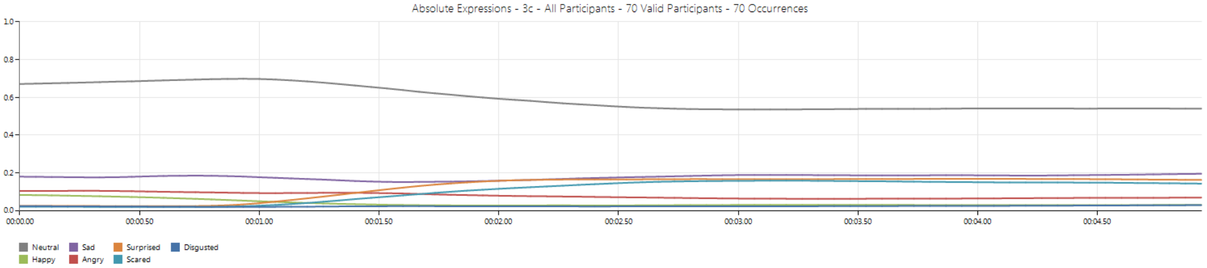

Figure A7. Intended Neutral Facial Expressions

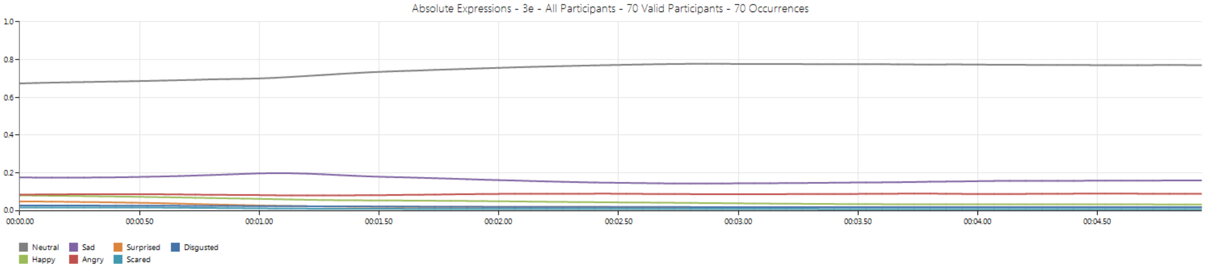

S2 Appendix

Machine Learning Procedure

Supplement B contains in depth information about the sequential steps in our machine learning procedure and includes additional details about the preprocessing, the hyperparameter tuning and the performance metrics. AU18 and AU27 showed zero variance in both datasets and were removed. Besides AU18 and AU27 we further removed all near zero variance predictors separately for the untrained participants and the trained actors (see Table B1), which met the following conditions: less than 20% unique values and a ratio between most frequent to second most frequent greater than 20. All remaining predictors underwent min-max normalization to reach a range from zero to one. We decided to restrict the multi-layer perceptrons to only one hidden-layer and used an extensive search to determine the optimal number of neurons in the single hidden-layer, which ranged from one to eighteen (M_d = 1.5). For the evaluation of the model performance, we used a grouped five-fold cross-validation. We used the grouped version of cross-validation to prevent emotions from one participant being allocated to the hold-out test set as well as the training data. In order to obtain the permutation importance of a predictor the baseline accuracy of the machine learning algorithm was compared with the accuracy after the values of one predictor were randomly shuffled (permuted). Permutation importance scores control per default for interaction effects, since associations between the predictors are destroyed during the shuffling of a predictor. We repeated the permutation 30 times for each predictor and averaged the resulting differences between the baseline accuracy and accuracy with one shuffled AU. This procedure allowed us to determine which AUs are relevant for the classification of a specific emotion category.

Table B1. Removed Near Zero Variance Action Units (AU) for Both Datasets

Category	Untrained Participants	Trained Actors
Joy	AU9, AU20	AU1, AU2, AU4, AU5, AU9, AU14, AU15, AU17, AU20, AU23, AU24, AU26, AU43
Anger	AU20	AU1, AU2, AU9, AU12, AU14, AU15, AU20, AU26, AU43
Surprise	AU9, AU20	AU4, AU6, AU7, AU9, AU10, AU12, AU14, AU15, AU17, AU20, AU23, AU24, AU43
Sadness	AU9, AU20	AU2, AU5, AU6, AU9, AU10, AU12, AU14, AU20, AU23, AU24, AU25, AU26, AU43
Disgust	-	AU1, AU2, AU5, AU12, AU14, AU15, AU20, AU23, AU24, AU26, AU43
Fear	AU9	AU6, AU9, AU10, AU12, AU14, AU15, AU17, AU23, AU24, AU43

S3 Appendix

FaceReader Emotion Scores

Table C shows Cohen's d for differences to zero (one-sample t-test against zero) for FaceReader (FR) emotion scores. For untrained participants strongest differences to zero can be reported for correct measures of intended joy, $t(68) = 48.75$, $p < .001$, $d = 5.87$, followed by sadness, $t(68) = 21.26$, $p < .001$, $d = 2.56$, surprise, $t(68) = 19.17$, $p < .001$, $d = 2.31$, anger, $t(68) = 18.00$, $p < .001$, $d = 2.17$, disgust, $t(68) = 14.07$, $p < .001$, $d = 2.17$, and lowest effects for fear, $t(68) = 9.83$, $p < .001$, $d = 1.18$. For trained actors, strongest differences to zero can also be reported for correct measures of intended joy, $t(66) = 112.82$, $p < .001$, $d = 13.78$, followed by surprise, $t(68) = 61.24$, $p < .001$, $d = 7.37$, disgust, $t(68) = 48.75$, $p < .001$, $d = 5.79$, sadness, $t(68) = 32.59$, $p < .001$, $d = 3.92$, anger, $t(68) = 24.11$, $p < .001$, $d = 2.90$, and again lowest effects for fear, $t(68) = 21.57$, $p < .001$, $d = 2.60$. It is noteworthy that in both datasets joy produced the strongest and fear the weakest effects.

Regarding measurement errors, no strong positive activation of an unintended FR measurement dimension can be reported for surprised, sad or disgusted faces. For intended scared faces, there is also a mismeasurement of FR surprise which is more pronounced for untrained participants, $t(68) = 8.63$, $p < .001$, $d = 1.04$, compared to the trained actors, $t(68) = 5.85$, $p < .001$, $d = 0.70$. For the trained actors a positive activation of FR sadness for angry faces, $t(68) = 7.05$, $p < .001$, $d = 0.85$, and of FR anger for happy faces can be observed, $t(66) = 6.69$, $p < .001$, $d = 0.82$. In summary, each emotion category is predicted correctly with large effects of the corresponding FR dimension in both datasets. Variation of these effects is more strongly pronounced in the untrained participants compared to the trained actors' datasets. Measurement errors are low for most emotion categories in both datasets in particular compared to the corresponding effect of the

aim dimension. One exception is the measurement of fearful faces for untrained participants which elicited comparable effects of FR fear and FR surprise.

Table C. Cohen's d for Differences to Zero for FaceReader (FR) Emotion Scores

Untrained Participants	Intended Emotion						
	Neutral	Joy	Anger	Surprise	Sadness	Disgust	Fear
FR Joy	-0.89	**5.87**	-0.68	-0.87	-1.02	-0.19	-0.21
FR Anger	-0.22	-0.86	**2.17**	-0.86	0.13	0.61	-0.24
FR Surprise	-0.37	-0.41	-0.70	**2.31**	-0.72	-0.66	**1.04**
FR Sadness	-0.28	-1.38	-0.18	-1.37	**2.56**	-0.55	-0.18
FR Disgust	-0.53	-0.10	0.18	-0.70	0.30	**1.69**	0.31
FR Fear	-0.50	-0.03	-0.42	0.73	-0.18	0.48	**1.18**

Trained Actors	Neutral	Joy	Anger	Surprise	Sadness	Disgust	Fear
FR Joy	**0.97**	**13.78**	0.19	0.24	0.33	0.44	0.22
FR Anger	0.70	**0.82**	**2.90**	0.44	0.49	0.69	0.33
FR Surprise	0.51	0.58	0.24	**7.37**	0.28	0.69	0.70
FR Sadness	**0.83**	0.35	0.85	0.38	**3.92**	0.50	0.35
FR Disgust	0.73	0.71	0.58	0.51	0.45	**5.79**	0.49
FR Fear	0.43	0.38	0.48	0.78	0.50	0.54	**2.60**

Note. Values indicate Cohen's d effect sizes in comparison to zero. Negative values reflect deactivation in comparison to zero. Bold values indicate large and positive effects (d > .8).

S4 Appendix

FaceReader (FR) Valence Scores.

Figure D. Mean FR Valence Scores separately for trained actors and untrained participants in arbitrary units [AU].

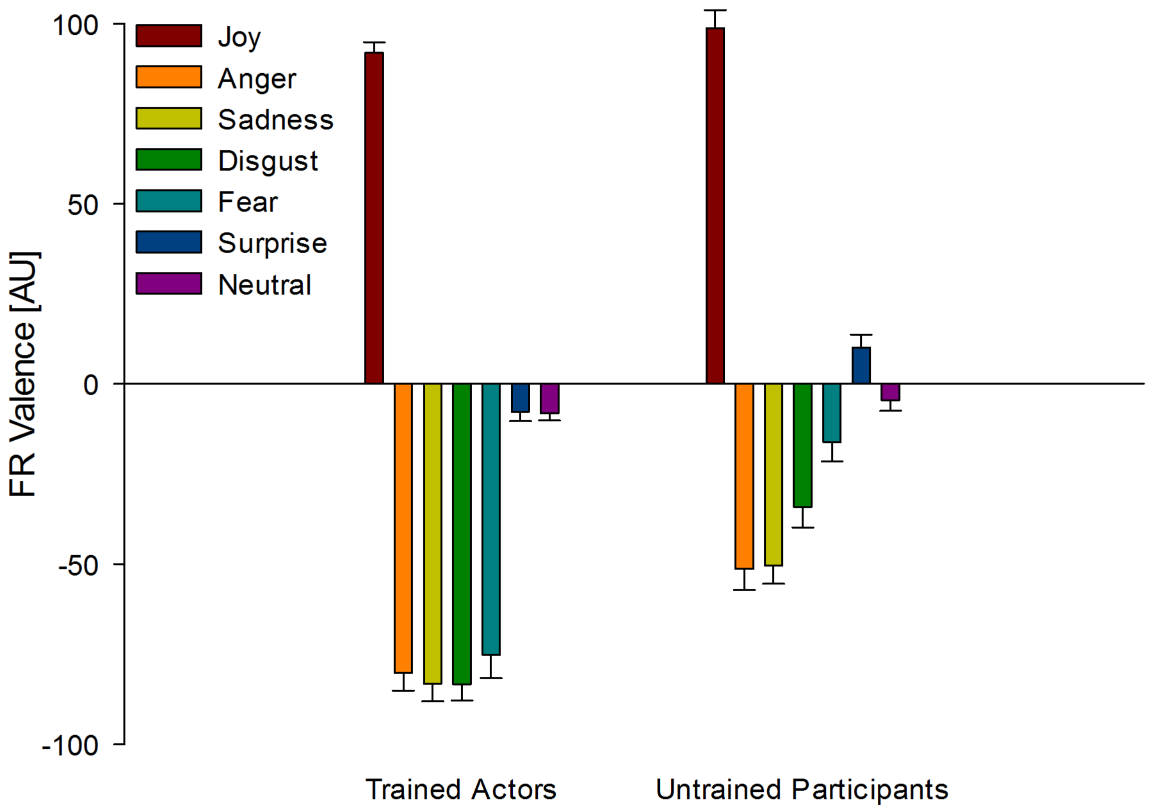

The colored bars indicate the different intended emotional facial expressions. Error bars indicate 95% confidence intervals. Positive values indicate pleasant facial expressions and negative values indicate unpleasant facial expressions.

Table D. Mean differences of Facereader Valence parameter for the emotion categories between datasets

Emotion Category	Untrained Participants *M*, (*SD*)	Trained Actors *M*, (*SD*)	*t*	*df*	*p*	*d*	Effect Inter-pretation
Joy	98.74 (21.26)	91.88 (12.27)	2.31	109.41	.023	.40	small
Anger	-51.37 (24.16)	-80.20 (20.50)	7.56	132.49	<.001	1.29	very large
Sadness	-50.42 (20.96)	-83.18 (20.64)	9.25	136.00	<.001	1.57	very large
Disgust	-34.19 (23.59)	-83.35 (18.64)	13.59	129.10	<.001	2.31	huge
Fear	-16.06 (22.34)	-75.15 (27.04)	14.00	136.00	<.001	2.38	huge
Surprise	10.09 (14.85)	-7.72 (10.87)	8.04	124.58	<.001	1.37	very large
Neutral	-4.55 (12.00)	-8.14 (8.15)	2.06	119.93	.042	.35	small

Note. t = t-values, df = corrected degrees of freedom, p = p-values, d = Cohen's d. M and SD represent mean and standard deviation. Positive values indicate pleasant facial expressions and negative values indicate unpleasant facial expressions. Standard deviation in parenthesis. $d \geq 0.2$ small; $d \geq 0.5$ medium; $d \geq 0.8$ large; $d \geq 1.2$ very large; $d \geq 2.0$ huge.

S5 Appendix

Cohen's d for Differences to Zero for FaceReader Action Units

Table E1 (Trained Actors) and Table E2 (Untrained Participants) show Cohen's d for differences to zero (one-sample t-test against zero) for FaceReader Action Units (AU). It is noteworthy that the most specific indicators regarding the different emotion categories were AU12 (Lip Corner Pull) for joy faces, AU26 (Jaw Drop) for surprise faces, AU09 (Nose Wrinkler) and AU10 (Upper Lip Raiser) for disgust faces, AU23 (Lip Tightener) and AU24 (Lip Pressor) for anger faces, AU15 (Lip Corner Depressor) for sadness faces and AU20 (Lip Stretcher) for fear faces. A large overlap of involved AU can be reported for surprised and scared faces: Both picture categories activated AU01, AU02, AU05 and AU25 which might be explanatory for the measurement confusion of FR emotion scores between the two categories described above. Furthermore, AU04 (Brow Lowerer) was coactivated unspecifically and with different effect sizes in all unpleasant facial expressions. AU25 was also unspecifically engaged in several expressions like joy, surprise, disgust and fear.

Table E1. Cohen's d for Differences to Zero for FaceReader Action Units for trained actors.

	Intended Emotions						
	Neutral	Joy	Anger	Surprise	Sadness	Disgust	Fear
AU01	0.20	0.15	0.27	**2.83**	**1.63**		**2.31**
AU02	0.24	0.15	0.15	**2.87**	0.21		**1.21**
AU04		0.16	**2.92**		**1.57**	**1.54**	**1.39**
AU05	0.12	0.12	**1.00**	**2.89**	0.23		**1.76**
AU06	0.23	**5.45**	0.48	0.18	0.24	**0.89**	0.23
AU07	0.22	0.53	**2.33**		0.41	**1.69**	0.29
AU09		0.20	0.25		0.12	**2.72**	
AU10		0.53	0.40	0.21	0.28	**2.49**	0.30
AU12		**14.68**	0.13		0.17	0.30	0.21
AU14			0.12	0.12	0.12	0.21	
AU15	0.12				**1.87**	0.26	0.12
AU17	0.16		**0.98**		**1.75**	0.59	0.17
AU18							
AU20	0.12		0.25	0.12	0.22	0.32	**1.06**
AU23			**1.26**		0.17	0.12	
AU24	0.16	0.17	**1.12**		0.12	0.12	
AU25	0.17	**4.32**	0.44	**1.93**	0.12	**1.57**	**3.02**
AU26		0.12	0.15	**3.09**			0.78
AU27				0.21			
AU43		0.12	0.16	0.12	0.12	0.16	

Note. Values indicate Cohen's d effect sizes in comparison to zero. Bold values indicate large and positive effects (d > .8). Empty cells indicate constant variables with M = 0 and SD = 0. AU01 = Inner Brow Raiser, AU02 = Outer Brow Raiser, AU04 = Brow Lowerer, AU05 = Upper Lid Raiser, AU06 = Cheek Raiser, AU07 = Lid Tightener, AU09 = Nose Wrinkler, AU10 = Upper Lid Raiser, AU12 = Lip Corner Pull, AU14 = Dimpler, AU15 = Lip Corner Depressor, AU17 = Chin Raiser, AU18 = Lip Puckerer, AU20 = Lip Stretcher, AU23 = Lip Tightener, AU24 = Lip Pressor, AU25 = Lips Part, AU26 = Jaw Drop, AU27 = Mouth Stretch, AU43 = Eyes Closed.

Table E2. Cohen's d for differences to zero for FaceReader Action Units for untrained participants.

	Intended Emotions						
	Neutral	Joy	Anger	Surprise	Sadness	Disgust	Fear
AU01	-0.19	-0.30	-0.18	**1.02**	0.63	0.26	**0.98**
AU02	-0.23	-0.14	-0.11	**1.19**	0.21	-0.08	0.70
AU04	-0.42	-0.40	**1.85**	-0.35	0.66	**1.35**	0.68
AU05	-0.19	-0.21	0.53	**0.97**	0.06	0.08	0.76
AU06	-0.34	**1.83**	-0.20	-0.20	-0.34	0.37	-0.16
AU07	-0.16	-0.16	**1.51**	-0.49	0.07	**1.36**	0.11
AU09	-0.18	-0.15	0.14	-0.01	-0.02	**1.08**	0.11
AU10	-0.18	-0.05	-0.11	-0.17	-0.14	**1.15**	0.26
AU12	-0.55	**4.69**	-0.34	-0.29	-0.54	0.01	0.00
AU14	-0.13	-0.26	0.33	-0.31	-0.13	-0.26	-0.25
AU15	0.05	-0.55	-0.36	-0.58	**1.65**	-0.31	-0.08
AU17	-0.05	-0.51	**0.99**	-0.51	**1.78**	0.30	-0.22
AU18	0.21	-0.13	0.17	0.11	0.17	-0.22	-0.18
AU20	-0.18	0.07	-0.13	-0.10	-0.16	0.38	0.66
AU23	0.17	-0.47	**1.60**	-0.50	0.34	-0.32	-0.41
AU24	-0.16	-0.44	**1.34**	-0.41	0.32	-0.11	-0.34
AU25	-0.43	**2.60**	-0.43	**1.88**	-0.48	**1.18**	**1.07**
AU26	-0.25	-0.23	-0.28	**1.47**	-0.27	0.02	0.40
AU27	-0.12			0.33			-0.11
AU43	-0.38	-0.48	-0.43	-0.47	0.07	-0.40	-0.50

Note. Values indicate Cohen's d effect sizes in comparison to zero. Negative values reflect deactivation in comparison to baseline. Bold values indicate large and positive effects (d > .8). Empty cells indicate constant variables with M = 0 and SD = 0. AU01 = Inner Brow Raiser, AU02 = Outer Brow Raiser, AU04 = Brow Lowerer, AU05 = Upper Lid Raiser, AU06 = Cheek Raiser, AU07 = Lid Tightener, AU09 = Nose Wrinkler, AU10 = Upper Lid Raiser, AU12 = Lip Corner Pull, AU14 = Dimpler, AU15 = Lip Corner Depressor, AU17 = Chin Raiser, AU18 = Lip Puckerer, AU20 = Lip Stretcher, AU23 = Lip Tightener, AU24 = Lip Pressor, AU25 = Lips Part, AU26 = Jaw Drop, AU27 = Mouth Stretch, AU43 = Eyes Closed.
